# ASO Author Reflections: Surgical Predictors for Survival in Patients Undergoing Distal Pancreatectomy for Pancreatic Ductal Adenocarcinoma

**DOI:** 10.1245/s10434-020-08911-x

**Published:** 2020-07-22

**Authors:** Maarten Korrel, Mohammad Abu Hilal, Marc G. Besselink

**Affiliations:** 1 Department of Surgery, Cancer Center Amsterdam, Amsterdam UMC, University of Amsterdam, Amsterdam, The Netherlands; 2 Department of General Surgery, Instituto Ospedaliero Fondazione Poliambulanza, Brescia, Italy

## Past

Distal pancreatectomy combined with systemic chemotherapy is the treatment of choice for (borderline) resectable left-sided pancreatic ductal adenocarcinoma (PDAC). Minimally invasive distal pancreatectomy has become the preferred approach for indications other than PDAC because of superior short-term outcomes.^1^,^2^ For PDAC specifically, standardized surgical methods have been described, including Gerota’s fascia resection and splenectomy, to optimize surgical radicality and adequate lymph node yield.^3^,^4^ Some studies have observed survival benefits when radical (R0) resection and adequate lymph node yield were obtained, but the prognostic significance of Gerota’s fascia resection and the minimally invasive approach remains understudied. This study retrospectively evaluated the impact of these surgical factors on overall survival after distal pancreatectomy for PDAC.

## Present

This multicenter retrospective study analyzed 1200 patients who underwent a distal pancreatectomy for PDAC from 34 centers in 12 countries of which 352 procedures (29%) were performed by a minimally invasive approach. Survival was assessed using Kaplan–Meier analysis. Cox proportional hazard analyses were performed to identify surgical predictors for survival. Median overall survival was 30 months [95% confidence interval (CI) 27–33], which was improved in patients with Gerota’s fascia resection [hazard ratio (HR) 0.74; 95% CI 0.57–0.95; *p* = 0.019], radical resection (HR 0.70; 95% CI 0.54–0.90; *p* = 0.006), and decreased lymph node ratio (HR 0.28; 95% CI 0.16–0.45; *p* < 0.001). Minimally invasive distal pancreatectomy did not worsen survival compared with open distal pancreatectomy (HR 1.14; 95% CI 0.87–1.49; *p* = 0.350). Excluding the 25% largest tumors from the analysis did not impair the prognostic significance of Gerota’s fascia resection. This study provided evidence that several surgical factors are associated with improved survival after distal pancreatectomy for PDAC, allowing the opportunity to integrate these into standard surgical practice to optimize oncological outcomes.^5^

## Future

With respect to the outcomes of this study, Gerota’s fascia resection should most likely be considered as a standardized step during distal pancreatectomy as it improves oncological outcomes such as surgical radicality and lymph node yield. However, the possible inherited biases of the retrospective nature of this study might have influenced outcomes, and these results should be confirmed in future, prospective studies to confirm their external validity. So far, studies in this patient group have been mostly retrospective cohort studies. Current ongoing randomized controlled trials, such as the DIPLOMA trial (ISRCTN44897265, www.e-mips.com/diploma-trial), may confirm these survival outcomes and the role of the minimally invasive approach during distal pancreatectomy for PDAC.
